# A validation study of crescents in predicting ESRD in patients with IgA nephropathy

**DOI:** 10.1186/s12967-018-1488-5

**Published:** 2018-05-03

**Authors:** Xiaoyan Zhang, Sufang Shi, Yan Ouyang, Meng Yang, Manman Shi, Xiaoxia Pan, Jicheng Lv, Zhaohui Wang, Hong Ren, Pingyan Shen, Weiming Wang, Hong Zhang, Jingyuan Xie, Nan Chen

**Affiliations:** 1 Department of Nephrology, Institute of Nephrology, Ruijin Hospital Affiliated with Shanghai Jiao Tong University School of Medicine, 197 Ruijin Er Road, Shanghai, 200025 China; 2 Renal Division, Peking University First Hospital, Institute of Nephrology, Key Laboratory of Renal Disease, Ministry of Health of China, Peking University, Beijing, China

**Keywords:** Crescent, IgA nephropathy (IgAN), Prognosis, End stage renal disease (ESRD)

## Abstract

**Background:**

A working group on the Oxford classification of IgA nephropathy (IgAN) recently reported that crescents detected in kidney tissue predicted a worse renal outcome. However, this finding must be validated in independent cohorts before it can be widely applied to clinical practice.

**Methods:**

Biopsy-proven IgAN patients were continuously recruited from two large renal centers in China from 1989 to 2014. All patients were followed for more than 1 year unless end stage renal disease (ESRD) occurred within 12 months. Crescents were defined as focal cellular or fibrocellular crescent formations. IgAN patients without detectable crescents were recruited to the C0 group. Patients with crescents in less than or more than 1/4 of all glomeruli were recruited to the C1 or C2 group, respectively. Primary outcome was defined as the time to ESRD, and the secondary outcome was defined as the time to an estimated glomerular filtration rate (eGFR) decline equal to or greater than 50% or to ESRD.

**Results:**

In total, 1152 IgAN patients were recruited in this study. Among all patients, 53.7% were in the C0 group, 38.8% were in the C1 group, and 7.5% were in the C2 group. Compared to patients in the C0 group, patients in the C1 or C2 group were younger, had more urinary protein excretion and lower eGFR, and presented with more severe mesangial hypercellularity, endocapillary proliferation or tubular atrophy/interstitial fibrosis. After 45 months of follow-up, ESRD had occurred in 80 (12.9%), 46 (10.3%) and 18 (20.9%) of patients in the C0, C1 and C2 groups, respectively. By multivariable Cox regression analysis, inclusion in the C1 (HR = 1.07, 95% CI 0.71–1.63), C2 (HR = 0.84, 95% CI 0.41–1.73), or C1 or C2 group (HR = 1.02, 95% CI 0.68–1.52) was not associated with a higher rate of ESRD than inclusion in the C0 group after adjusting for age, gender, eGFR, mean arterial pressure (MAP), MEST scores, and immunosuppressive treatment. However, in patients with nephrotic-range proteinuria, patients in either the C1 or C2 group had a higher rate of the primary outcome, ESRD (HR = 2.54, 95% CI 1.14–5.66) after adjusting for age, gender, eGFR, MAP, MEST scores, and immunosuppressive treatment. Similar results were found when we evaluated the association between crescents and the secondary outcome.

**Conclusions:**

IgAN patients with crescents had more severe clinical and pathological manifestations than those without crescents. However, we failed to replicate the association between crescents and renal function progression in Chinese IgAN patients followed for more than 1 year.

**Electronic supplementary material:**

The online version of this article (10.1186/s12967-018-1488-5) contains supplementary material, which is available to authorized users.

## Background

IgA nephropathy (IgAN) is the most common primary glomerulonephritis worldwide and is characterized by prominent IgA1 deposition in the glomerular mesangium [1, 2]. The clinical course of IgAN and associated pathological lesions are highly variable [3–6]. Crescent formation is a common histopathological finding, occurring in approximately 20–50% of IgAN patients [7–11]. However, crescents were not effectively evaluated in the Oxford scoring system, the most widely accepted pathological classification of IgAN, nor in subsequent validation studies of the Oxford classification, including two large Chinese cohort studies [9, 12–14]. In addition, the value of crescents in predicting renal function progression in IgAN patients is inconsistent among patients of different ethnicities. Recently, crescents were reported to predict a worse renal outcome by an Oxford classification working group [15]. Based on 3096 IgAN patients from 16 countries across the world, crescents were found to be associated with a composite outcome including either ESRD or a 50% reduction in estimated glomerular filtration rate (eGFR) in patients not receiving immunosuppression. Furthermore, crescents in more than 1/4 of glomeruli remained a predictor of poor outcomes in patients receiving immunosuppression. However, the inconsistency in the association between crescents and renal function deterioration among all cohorts in the study, for example, the Chinese cohort, suggested heterogeneity may exist among different populations. Therefore, the finding of this study needs to be validated in independent cohorts, especially in the Chinese population. In this study, we aim to evaluate the predictive value of crescents based on two extended Chinese IgAN cohorts.

## Methods

### Study population and design

We continuously recruited idiopathic IgAN patients from two extended renal centers in China (Department of Nephrology, Ruijin Hospital, affiliated with Shanghai Jiao Tong University School of Medicine; and Renal Division, Department of Medicine, Peking University First Hospital) between 1989 and 2014. The inclusion criteria were as follows: (1) Patients were diagnosed with primary IgAN by renal biopsy; (2) Patients were followed for more than 12 months unless ESRD occurred within 12 months; and (3) Patients did not have systemic diseases, such as systemic lupus erythematosus, Henoch–Schonlein purpura, or liver cirrhosis. Renal tissue slides were reviewed and scored by two experienced renal pathologists, and at least 8 glomeruli per slide were required for further review. A crescent was defined as an extracapillary lesion of any size containing more than two cell layers. A cellular crescent was indicated when more than 50% of the lesion was occupied by cells, and a fibrocellular crescent was defined as an extracapillary lesion comprising cells and extracellular matrix, with less than 50% cells and less than 90% matrix. A fibrous crescent was defined as a lesion composed of more than 90% matrix covering the circumference of Bowman’s capsule, and patients with these crescents were excluded from this study. Patients with crescents in < 1/4 of all glomeruli were assigned to the C1 group. Patients with crescents in ≥ 1/4 of all glomeruli were assigned to the C2 group, and patients without crescents were assigned to the C0 group. Baseline demographic and clinical data, including age, gender, mean arterial pressure (MAP), 24-h protein excretion and estimated glomerular filtration rate (eGFR) calculated by the CKD-EPI equation, were collected from all patients at the time of renal biopsy [16]. Nephrotic-range proteinuria was defined as 24-h urine protein ≥ 3.5 g/24 h. Hypertension was defined as systolic blood pressure ≥ 140 mmHg (18.7 kPa) and/or diastolic blood pressure ≥ 90 mmHg (12.0 kPa) or the use of antihypertensive agents at the time of renal biopsy. Immunosuppressive therapy was defined as treatment with corticosteroids and/or corticosteroid-sparing agents (including cyclophosphamide, azathioprine, mycophenolate, cyclosporine or tacrolimus). All biopsy specimens were processed routinely using standard light microscopy, immunofluorescence (IF) and electron microscopy (EM). For each biopsy specimen, the degrees of mesangial hypercellularity, endocapillary hypercellularity, segmental glomerulosclerosis and tubular atrophy/interstitial fibrosis were evaluated semi-quantitatively according to the Oxford Classification of IgAN. M1 was defined as a mesangial hypercellularity score higher than 0.5, E1 was defined as the presence of endocapillary hypercellularity, S1 was defined as the presence of segmental glomerulosclerosis, T1 was defined as tubular atrophy/interstitial fibrosis within 26–50% of the cortical area, and T2 was defined as tubular atrophy/interstitial fibrosis in greater than 50% of the cortical area. The primary outcome was the time to ESRD, defined as eGFR < 15 ml/min/1.73 m^2^ and the need for renal replacement therapy (dialysis or renal transplantation). The secondary outcome was the time to an eGFR decline of at least 50% or the time to ESRD, which was evaluable in 957 patients.

This study adhered to the principles of the Declaration of Helsinki II and was approved by the Institutional Review Board of Ruijin Hospital, Shanghai Jiao Tong University School of Medicine [Clinical Trial Ethics Committee Approval (2012-38)].

### Statistical analyses

Categorical variables are presented as the number (percentage); quantitative variables were assessed for normality, and these data are presented as the mean ± SD (or median and interquartile range for non-normally distributed variables). Normally distributed continuous data were compared across groups using the independent sample t test or one-way ANOVA. Non-normally distributed continuous data were compared across groups using the Mann–Whitney U test or Kruskal–Wallis test. Categorical variables are presented as the frequency or percentage (%), and proportions were compared using Pearson’s χ^2^ test or Fisher’s test as appropriate. Renal survival was measured from the time of biopsy and was analyzed using the Kaplan–Meier method, and the equality of survival functions was examined using the log-rank test. Multivariate Cox regression analysis or linear regression was applied to identify independent factors associated with renal outcomes and eGFR slope (P < 0.05 indicated statistical significance). Statistical analyses were performed using SPSS (version 13.0).

## Results

### Characteristics of validation patients

We recruited a total of 1152 patients, of which 566 (49.1%) were male, and the mean age was 35 ± 12 years. At the time of renal biopsy, patients had a urinary protein excretion of 1.4 (0.7–2.6) g/24 h and an eGFR of 79.1 (52.1–101.7) ml/min/1.73 m^2^. The mean MAP was 95.8 ± 13.5 mmHg, and 33.1% (381) of patients presented with hypertension. In total, 533 (46.3%) patients had crescents in glomeruli. Of these patients, 447 (38.8%) had crescents in less than 1/4 of glomeruli (C1 group), and 86 (7.5%) had crescents in more than 1/4 of glomeruli (C2 group). Regarding MEST Oxford scores in all patients, 43.1% were M1, 42.1% were E1, 77.0% were S1, and 33.4% were T1/T2. Compared to the C0 group, patients in the C1 and C2 groups were younger (34.3 ± 11.9 years in C1 and 33.0 ± 10.6 years in C2 vs 36.5 ± 12.1 years in C0) and had higher levels of proteinuria [1.4 (0.8–2.7) g/24 h in C1 and 2.5 (1.6–4.0) g/24 h in C2 vs 1.2 (0.6–2.4) g/24 h in C0]. Patients in the C2 group had a lower eGFR than those in the C0 group [66.0 (47.1–92.0) vs 81.7 (56.6–100.8) ml/min/1.73 m^2^].

Compared to the C0 group, the C1 and C2 groups had more patients classified as M1 (51.1% in C1 and 56.4% in C2 vs 35.5% in C0) and E1 (56.2% in C1 and 80.8% in C2 vs 26.8% in C0). Meanwhile, there were more S1 patients in the C1 group than in the C0 group (85.4% vs 72.0%), and more T1/T2 cases were found in the C2 group than in the C0 group (46.2% vs 33.3%). Immunosuppressive agents, including corticosteroids with or without corticosteroid-sparing agents, were more commonly used in the C2 group than in the C0 group (81.0% vs 49.8%) (Table 1). Finally, among all recruited patients, the primary outcome of ESRD occurred in 144 individuals (12.5%), and the secondary outcome occurred in 162 individuals (14.1%).

**Table 1:** Baseline cohort characteristics

Variables	Overall	C0	C1	C2
	(n = 1152)	(n = 619)	(n = 447)	(n = 86)
Follow-up (months)	45 (25–70)	45 (24–70)	43 (26–68)	59 (27–88)
eGFR decline rate (ml/min/1.73 m^2^/year)	− 1.1 (− 4.6 to − 1.8)	− 1.2 (− 4.6 to − 1.6)	− 1.0 (− 4.3 to − 2.0)	− 0.25 (− 4.6 to − 3.43)
Male (%)	566 (49.1)	304 (49.1)	221 (49.4)	41 (47.7)
Age (years)	35.4 ± 12.0	36.5 ± 12.1	34.3 ± 11.9*	33.0 ± 10.6*
Urinary protein (g/d)	1.42 (0.72–2.62)	1.19 (0.62–2.39)	1.44 (0.82–2.71)*	2.51 (1.62–3.99)**
eGFR (ml/min/1.73 m^2^)	79.12 (52.08–101.67)	78.46 (48.37–103.41)	81.67 (56.59–100.82)	65.95 (47.11–91.95)*
Hypertension (%)	381 (33.1)	217 (35.1)	134 (30.0)	30 (34.9)
MAP (mmHg)	95.8 ± 13.5	96.2 ± 13.3	94.9 ± 13.3	97.6 ± 16.2
Oxford classification				
M1 (%)	469/1089 (43.1)	208/586 (35.5)	217/425 (51.1)**	44/78 (56.4)**
E1 (%)	459/1089 (42.1)	157/586 (26.8)	239/425 (56.2)**	63/78 (80.8)**
S1 (%)	839/1089 (77.0)	422/586 (72.0)	363/425 (85.4)**	54/78 (69.2)
T1 + T2 (%)	364/1089 (33.4)	195/586 (33.3)	133/425 (31.3)	36/78 (46.2)*
With immunosuppression (%)	566/1078 (52.5)	288/578 (49.8)	210/416 (50.5)	68/84 (81.0)**

*ESRD* end stage renal disease, *eGFR* estimated glomerular filtration rate, *MAP* mean arterial pressure

* P < 0.05 and ** P < 0.001 compared to the C0 group

### Crescent formation and renal outcomes

**Fig. 1:**
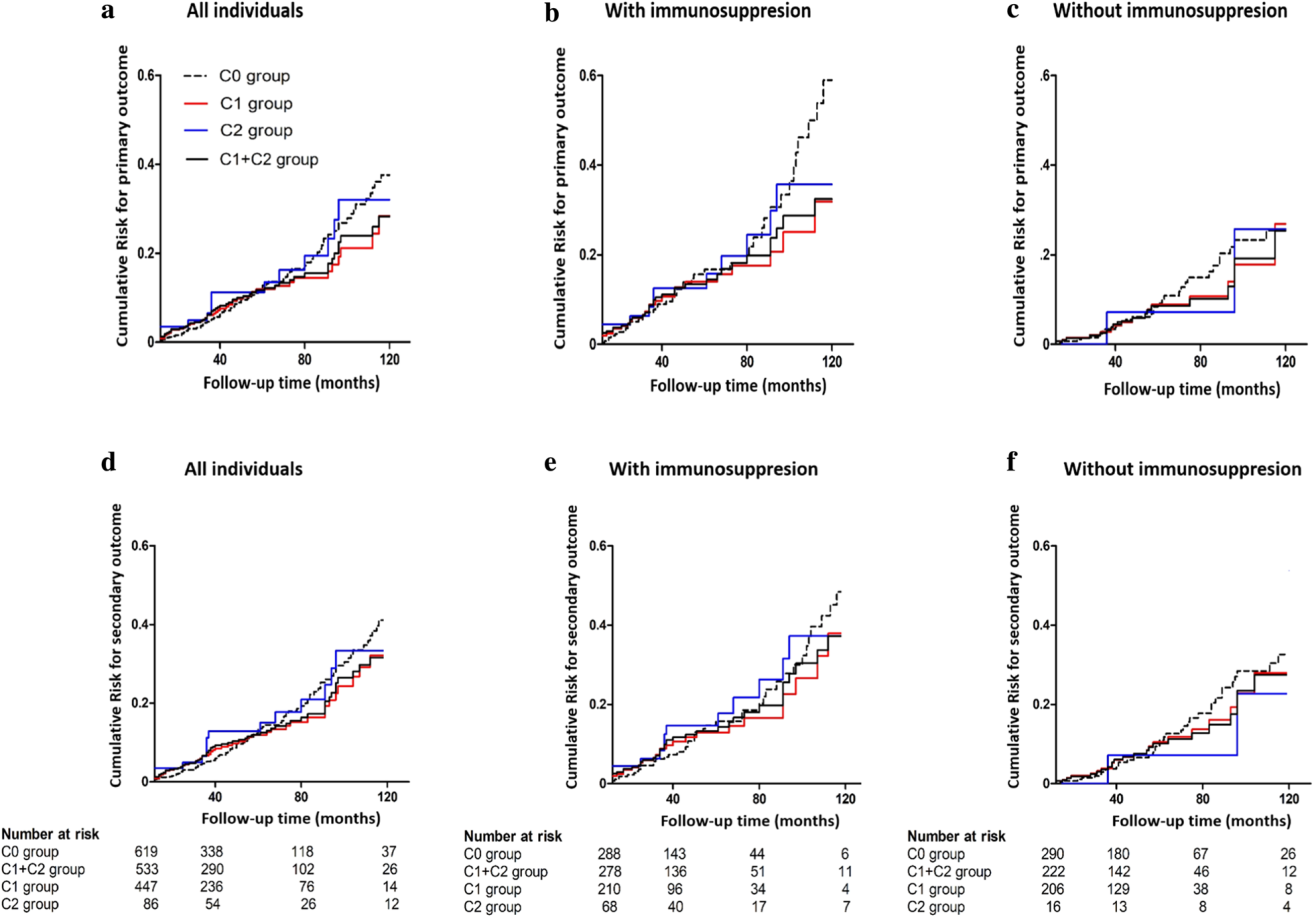
Kaplan–Meier curves of the cumulative risk of the primary outcome for patients in each crescent group: all patients (**a**), those with immunosuppression (**b**), and those without immunosuppression (**c**). Kaplan-Meier curves of the cumulative risk of the secondary outcome for patients in each crescent group: all patients (**d**), those with immunosuppression (**e**), and those without immunosuppression (**f**). The mean primary outcome-free survival times for the C0, C1, C2, and C1 + C2 groups were 176.3 ± 7.5, 225.4 ± 14.8, 168.9 ± 7.3 and 187.5 ± 18.6 months (P_*C1* vs *C0*_ = 0.45, P_*C2* vs *C0*_ = 0.37, P_*C1*+*C2* vs *C0*_ = 0.82) for all patients; 146.3 ± 13.4, 132.6 ± 6.2, 134.2 ± 11.8 and 139.4 ± 8.4 (P_*C1* vs *C0*_, = 0.27, P_*C2* vs *C0*_ = 0.77, P_*C1*+*C2* vs *C0 *_= 0.39) for patients with immunosuppression; and 190.4 ± 9.6, 225.1 ± 23.0, 151.9 ± 23.5 and 188.1 ± 25.4 months (P_*C1* vs *C0*_ = 0.66, P_*C2* vs *C0*_ = 0.59, P_*C1*+*C2* vs *C0*_ = 0.87) for patients without immunosuppression, respectively. The mean secondary outcome-free survival times for the C0, C1, C2, and C1 + C2 groups were 163.7 ± 7.8, 188.9 ± 18.3, 140.9 ± 11.1 and 171.7 ± 16.3 months (P_*C1* vs *C0 *_= 0.73, P_*C2* vs *C0*_ = 0.52, P_*C1*+*C2* vs *C0*_ = 0.98) for all patients; 138.6 ± 14.0, 117.0 ± 8.1, 131.8 ± 11.7 and 130.0 ± 8.1 months (P_*C1* vs *C0*_, = 0.60, P_*C2* vs *C0*_ = 0.93, P_*C1*+*C2* vs *C0*_ = 0.67) for patients with immunosuppression; and 171.9 ± 10.2, 197.8 ± 25.6, 151.9 ± 23.5 and 172.7 ± 22.1 months (P_*C1* vs *C0*_ = 0.81, P_*C2* vs *C0*_ = 0.94, P_*C1*+*C2* vs *C0*_ = 0.89) for patients without immunosuppression, respectively

Kaplan–Meier curves showed that patients in the C0, C1 and C2 groups had a similar mean primary outcome-free time (176.3 ± 7.5, 225.4 ± 14.8 and 168.9 ± 7.3 months) and mean secondary outcome-free time (163.7 ± 7.8, 188.9 ± 18.3 and 140.9 ± 11.1 months). The multivariable Cox regression model indicated that patients in the C1 (HR = 1.07, 95% CI 0.71–1.63) and C2 groups (HR = 0.84, 95% CI 0.41–1.73) did not have a higher risk of ESRD than patients in the C0 group. Similarly, patients in the C1 (HR = 1.01, 95% CI 0.69–1.48) and C2 groups (HR = 0.77, 95% CI 0.40–1.50) did not have a higher risk of the secondary outcome than those in the C0 group after adjusting for age, gender, eGFR, MAP, MEST scores, and immunosuppression (Fig. 1, Table 2). Furthermore, patients in the C0, C1 and C2 groups had a similar eGFR decline rate [− 1.2 (− 4.6 to − 1.6) ml/min/1.73 m^2^/year, − 1.0 (− 4.3 to − 2.0) ml/min/1.73 m^2^/year and − 0.25 (− 4.6 to − 3.43) ml/min/1.73 m^2^/year] (Table 1, Additional file 1: Table S2).

**Table 2:** Multivariate analysis of crescents with renal outcomes in IgAN patients with or without immunosuppression

Variable	All patients			With immunosuppression		Without immunosuppression	
	Events	HR (95% CI)	P value	HR (95% CI)	P value	HR (95% CI)	P value
Primary outcome	144 (12.5)						
C0 (n = 619)	80 (12.9)	Reference	/	Reference	/	Reference	/
C1 (n = 447)	46 (10.3)	1.07 (0.71–1.63)	0.74	0.76 (0.42–1.40)	0.38	1.05 (0.53–2.08)	0.89
C2 (n = 86)	18 (20.9)	0.84 (0.41–1.73)	0.63	1.09 (0.41–2.86)	0.87	0.48 (0.12–1.91)	0.30
C1 + C2 (n = 533)	64 (12.0)	1.02 (0.68–1.52)	0.93	0.85 (0.49–1.49)	0.58	0.88 (0.46–1.70)	0.71
Secondary outcome	162 (14.1)						
C0 (n = 619)	89 (14.4)	Reference	/	Reference	/	Reference	/
C1 (n = 447)	54 (12.1)	1.01 (0.69–1.48)	0.97	0.80 (0.46–1.41)	0.44	0.88 (0.49–1.60)	0.68
C2 (n = 86)	19 (22.1)	0.77 (0.40–1.50)	0.44	1.16 (0.49–2.73)	0.74	0.32 (0.09–1.21)	0.09
C1 + C2 (n = 533)	73 (13.7)	0.93 (0.64–1.33)	0.68	0.86 (0.51–1.45)	0.57	0.72 (0.41–1.29)	0.27

HRs were adjusted for age, gender, initial eGFR, MAP, proteinuria, and Oxford classification indicators (including mesangial hypercellularity, endocapillary hypercellularity, segmental glomerulosclerosis and tubular atrophy/interstitial fibrosis)

### Subgroup analyses for crescent formation and progression to ESRD

**Fig. 2:**
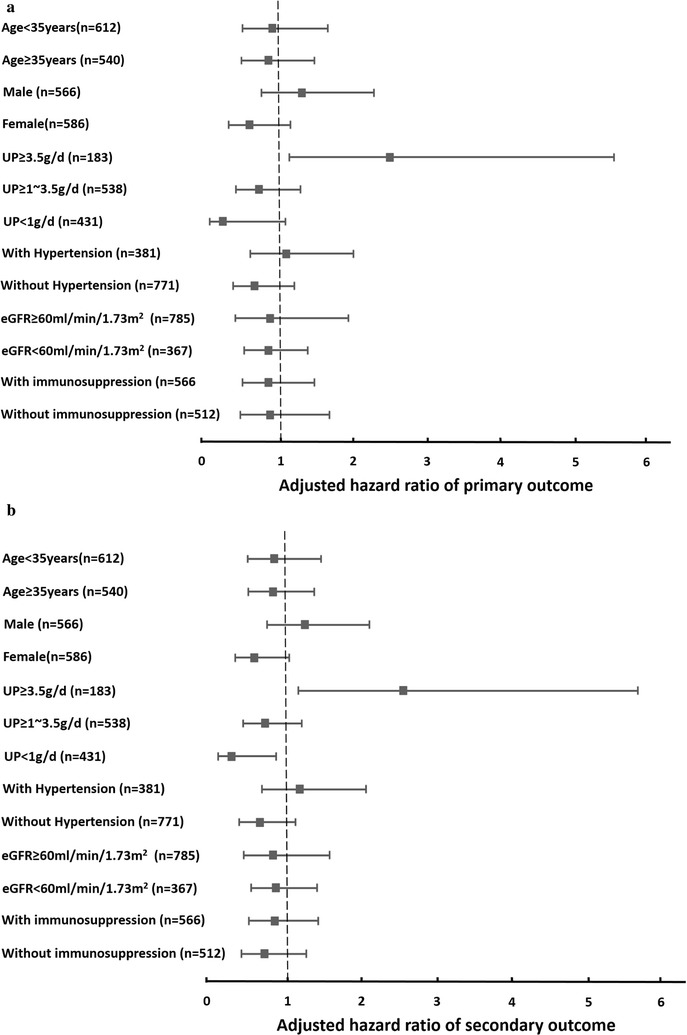
Stratified Cox regression models of the association of crescents with the primary outcome (**a**) and secondary outcome (**b**) in each subgroup. HRs were adjusted by initial eGFR, MAP, proteinuria, Oxford classification indicators (including mesangial hypercellularity, endocapillary hypercellularity, segmental glomerulosclerosis and tubular atrophy/interstitial fibrosis), and immunosuppressive therapy. *UP* urinary protein, *eGFR* estimated glomerular filtration rate

All patients were subdivided into subgroups based on the main clinical characteristics or whether they were receiving immunosuppressive agents. In patients with nephrotic-range proteinuria, crescent formation was associated with an increased risk of the primary outcome (HR = 2.54, 95% CI 1.14–5.66, P = 0.02) and secondary outcome (HR = 2.61, 95% CI 1.18–5.80, P = 0.02) after adjusting for age, gender, eGFR, MAP, pathological indicators, and immunosuppression. No associations between crescent formation and the primary or secondary outcome were found in the other subgroups, including those based on age, gender, blood pressure, eGFR and immunosuppressive agents (Fig. 2).

## Discussion

A recently study conducted by Haas and colleagues examined the presence of crescents in kidney tissue from IgAN patients and evaluated the effect of crescents on renal function progression [15]. In their multi-center retrospective cohort of 3096 IgAN patients [11–13, 17], crescents were found to be associated with an increased risk of renal outcomes (defined as a combination of ESRD and a 50% eGFR decline), especially in patients not using immunosuppressive agents when 1/4 was set as the cut-off for categorizing crescents. However, this finding must be validated in independent cohorts with diverse populations. In this study, we aimed to validate the association between crescents and IgAN progression in an extended Chinese cohort of IgAN patients who were followed for at least 1 year. Not surprisingly, we found that patients with crescents in kidney tissue had more severe clinical and pathological manifestations. However, we failed to validate crescents as an independent predictor of ESRD after adjusting for clinical and pathological variables in IgAN patients with or without immunosuppressive treatment, except in patients with nephrotic-range proteinuria.

Crescents are a common lesion identified by renal biopsy in IgAN patients and indicate severe glomerular injury. IgAN patients with crescentic glomerulonephritis (crescents in more than 50% of glomeruli) are recommended to undergo more aggressive treatment such as steroids or immunosuppressive agents according to the KDIGO guidelines [18]. However, the treatment strategy for IgAN patients with crescents in fewer than 50% of glomeruli is less clear, and the prognosis of these patients is uncertain since studies in this field have not reported consistent results [10, 19–24]. For example, neither the original Oxford study nor its largest validation study, the VALIGA study, found an association between crescents and the risk of renal function progression in IgAN patients [12, 17]. In contrast, the presence of crescents conveyed a 2.3-fold increased risk of renal failure in a meta-analysis that pooled 1487 IgAN patients from four Asian studies and one European study. The largest study in this field recruited 3096 patients from four large cohorts, including those in the original Oxford study and the VALIGA study. In this study, patients with any degree of crescents were included, and the associations between crescents and renal outcomes were evaluated by using 25% as the cut-off point of crescent formation, which was suggested by a previous study. However, we failed to replicate the association between crescent formation and renal outcomes, including the single outcome of ESRD and the combined outcome of a 50% eGFR decline and ESRD. Possible explanations include the different formulas used by the studies to estimate eGFR. The study by Hass used the four-variable Modification of Diet in Renal Disease (MDRD) formula, whereas we used the CKD-EPI formula, which might be more accurate than the MDRD formula. Similar to our result, Lee et al. reported findings for 430 Korean IgAN patients after using the CKD-EPI formula to evaluate the prognostic value of crescents. Their results showed that crescents were not an independent prognostic factor by multivariable Cox analysis after adjusting for clinical factors and Oxford classification [25]. The different definitions of outcomes may also partially account for the discrepant findings. The study by Haas used a combined endpoint of a 50% decline in eGFR and ESRD, whereas other studies have used ESRD, which is a more robust outcome. However, considering the intrinsic nature of crescents, some IgAN patients with more crescents may present at an acute and early stage of renal damage. Consequently, the renal function decline in these patients may be reversible, even if the rate of eGFR decrease is more than 50%. In this study, we failed to validate the increased risk of renal function progression in Chinese IgAN patients in the C1 or C2 groups compared to those in the C0 group by using different definitions of renal outcomes. In addition, we used a cut-off of one-sixth to increase the fraction of patients with crescents, and the results remained negative (Additional file 1: Table S1). Heterogeneity exists among the four cohorts included in the study by Haas [15]. Crescents were not associated with renal survival in the original Chinese study by CH Zeng et al. [13]. Furthermore, in the Japanese cohort including 702 IgAN patients, the predictive value of crescents was evident only in patients with initial proteinuria < 0.5 g/day and an eGFR < 30 ml/min/1.73 m^2^, whereas it was not seen in patients who met the inclusion criteria of the Oxford classification [11]. Thus, it is possible that crescents have better predictive value in severe cases of IgAN.

Although we were unable to replicate the association between crescents and ESRD in the whole cohort, subgroup analyses revealed that crescents were associated with a higher risk of ESRD in patients with nephrotic-range proteinuria. Proteinuria ≥ 1 g/day has been identified as a risk factor for unfavorable prognosis in IgAN [26]. The above result indicated a synergistic effect between proteinuria and crescent formation. An earlier study found that widespread alterations in podocytes, including foot process effacement and prominent microvillous transformation, were observed in the process of crescent formation. Podocytes further initiated the proliferation of parietal epithelial cells to form cellular crescents by adhering to both the glomerular basement membrane and the parietal basement membrane [27, 28]. The evidence for podocytes being involved in the pathogenesis of crescent formation provides further clues regarding the association between crescent formation and the development of proteinuria.

There were limitations to this study. First, due to the retrospective study design, we could not evaluate crescent size, which possibly influences renal survival. Additionally, only 86 (7.5%) patients were in the C2 group; this relatively low frequency of patients in the high crescent group might have decreased the statistical power in analyzing the clinical impact.

## Conclusions

In this study, we found that IgAN patients with crescent formation had more severe clinical and pathological manifestations. However, we failed to replicate the association between crescents and renal function progression in Chinese IgAN patients with at least 1 year of follow-up.

## Additional file

**Additional file 1: Table S1.** Adjusted HRs of the association of ESRD with crescents in more than 1/6 of glomeruli. **Table S2.** Univariate analysis of crescents with eGFR decline rate (ml/min/1.73 m^2^/year).

## Abbreviations

IgAN: IgA nephropathy
ESRD: end stage renal disease
eGFR: estimated glomerular filtration rate
MAP: mean arterial pressure
UP: urinary protein
