# Supplementary material for: A validation study of crescents in predicting ESRD in patients with IgA nephropathy
Source: J Transl Med. 2018 May 3;16:115. doi: 10.1186/s12967-018-1488-5 (PMC5934890; doi:10.1186/s12967-018-1488-5)
Supplement: Supplementary file 1 — Additional file 1: Table S1. Adjusted HRs of the association of ESRD with crescents in more than 1/6 of glomeruli. Table S2. Univariate analysis of crescents with eGFR decline rate (ml/min/1.73 m2/year). [file 12967_2018_1488_MOESM1_ESM.docx]

Table S1. Adjusted HRs of the association of ESRD with crescents in more than 1/6 of glomeruli

| **Variable** | **All Patients** |  | **With immunosuppression** |  | **Without immunosuppression** |  |
| --- | --- | --- | --- | --- | --- | --- |
|  | **HR (95% CI)** | **P value** | **HR (95% CI)** | **P value** | **HR (95% CI)** | **P value** |
| No Crescent group (n=619) | Reference | / | Reference | / | Reference | / |
| Crescent group(n=533) | 1.02 (0.68-1.52) | 0.93 | 0.85 (0.49-1.49) | 0.58 | 0.88 (0.46-1.70) | 0.71 |
| <1/6 (n=386) | 1.09 (0.70-1.69) | 0.70 | 0.81 (0.41-1.58) | 0.53 | 1.10 (0.61-2.41) | 0.79 |
| ≥1/6 (n=147) | 0.89 (0.48-1.67) | 0.72 | 1.06 (0.47-2.40) | 0.89 | 0.42 (0.12-1.49) | 0.18 |

Note: HRs were adjusted for age, gender, initial eGFR, MAP, proteinuria, and the Oxford classification indicators (including mesangial hypercellularity, endocapillary hypercellularity, segmental glomerulosclerosis and tubular atrophy/interstitial fibrosis)

Table S2. Univariate analysis of crescents with eGFR decline rate (ml/min/1.73 m^2^/year)

| **Variable** | **All Patients** |  | **With immunosuppression** |  | **Without immunosuppression** |  |
| --- | --- | --- | --- | --- | --- | --- |
|  | **β coefficient (95% CI)** | **P value** | **β coefficient**  **(95% CI)** | **P value** | **β coefficient**  **(95% CI)** | **P value** |
| C0 (n=619) | Reference | / | Reference | / | Reference | / |
| C1 (n=447) | 0.02 (-0.80 to -1.68) | 0.70 | 0.04 (-1.17 to -2.82) | 0.42 | -0.03 (-1.86 to -0.94) | 0.52 |
| C2 (n=86) | 0.03 (-0.79 to -1.84) | 0.43 | 0.02 (-1.36 to -2.06) | 0.69 | 0.03 (-1.77 to -2.94) | 0.62 |
| C1+C2 (n=533) | 0.03 (-0.69 to -1.74) | 0.40 | 0.04 (-1.12 to -2.72) | 0.42 | -0.03 (-1.75 to -0.99) | 0.59 |
